# A unique antigen against SARS-CoV-2, *Acinetobacter baumannii,* and *Pseudomonas aeruginosa*

**DOI:** 10.1038/s41598-022-14877-5

**Published:** 2022-06-27

**Authors:** Mohammad Reza Rahbar, Shaden M. H. Mubarak, Anahita Hessami, Bahman Khalesi, Navid Pourzardosht, Saeed Khalili, Kobra Ahmadi Zanoos, Abolfazl Jahangiri

**Affiliations:** 1 Pharmaceutical Sciences Research Center, Shiraz University of Medical Sciences, Shiraz, Iran; 2 Department of Clinical Laboratory Science, Faculty of Pharmacy, University of Kufa, Najaf, Iraq; 3 School of Pharmacy, Shiraz University of Medical Sciences, Shiraz, Iran; 4 Department of Research and Production of Poultry Viral Vaccine, Razi Vaccine and Serum Research Institute, Agricultural Research Education and Extension Organization, Karaj, Iran; 5 Biochemistry Department, Guilan University of Medical Sciences, Rasht, Iran; 6 Department of Biology Sciences, Shahid Rajaee Teacher Training University, Tehran, Iran; 7 Young Researchers Club, Science and Research Branch, Islamic Azad University, Tehran, Iran; 8 Applied Microbiology Research Center, Systems Biology and Poisonings Institute, Baqiyatallah University of Medical Sciences, Vanak Sq. Molasadra St., P.O. Box 1435915371, Tehran, Iran

**Keywords:** Computational biology and bioinformatics, Immunology, Microbiology

## Abstract

The recent outbreak of COVID-19 has increased hospital admissions, which could elevate the risk of nosocomial infections, such as *A. baumannii* and *P. aeruginosa* infections. Although effective vaccines have been developed against SARS-CoV-2, no approved treatment option is still available against antimicrobial-resistant strains of *A. baumannii* and *P. aeruginosa.* In the current study, an all-in-one antigen was designed based on an innovative, state-of-the-art strategy. In this regard, experimentally validated linear epitopes of spike protein (SARS-CoV-2), OmpA (*A. baumannii*), and OprF (*P. aeruginosa*) were selected to be harbored by mature OmpA as a scaffold. The selected epitopes were used to replace the loops and turns of the barrel domain in OmpA; OprF_311–341_ replaced the most similar sequence within the OmpA, and three validated epitopes of OmpA were retained intact. The obtained antigen encompasses five antigenic peptides of spike protein, which are involved in SARS-CoV-2 pathogenicity. One of these epitopes, viz. QTQTNSPRRARSV could trigger antibodies preventing super-antigenic characteristics of spike and alleviating probable autoimmune responses. The designed antigen could raise antibodies neutralizing emerging variants of SARS-CoV-2 since at least two epitopes are consensus. In conclusion, the designed antigen is expected to raise protective antibodies against SARS-CoV-2, *A. baumannii,* and *P. aeruginosa*.

## Introduction

A recent pandemic caused by a novel coronavirus known as respiratory syndrome coronavirus 2 (SARS-CoV-2) has caught the international community by surprise. Until 19 January 2022, 332,617,707 confirmed human infections and 5,551,314 confirmed deaths have been reported (https://covid19.who.int/). Several strategies^1–4^ such as immunization had been suggested to retard the rapid spread of SARS-CoV-2. Despite the vast vaccination programs, the pandemic is continuing, nevertheless, mass immunization has represented a key intervention for palliation of the disease severity and transmission^5^.

Although the mortality rate of this virus is low (3–4%), SARS-CoV-2 could rapidly decimate and lead to a high rate of hospitalization due to its high speed of propagation^6,7^. Hospital admission could be the initiation of a tragedy. Coronavirus disease 2019 (COVID-19) could put the patients, particularly those with severe pneumonia, at the risk of notorious nosocomial pathogens such as *Acinetobacter baumannii* and *Pseudomonas aeruginosa*. Several reports have highlighted the possible outbreak of nosocomial infections concurrent with a high rate of hospital admission due to COVID-19^8–11^. *A. baumannii* and *P. aeruginosa* are among the most successful nosocomial pathogens with respective mortality rates of up to 70%^12^ and 61%^13^. World Health Organization (WHO) has considered these bacteria as priority 1 (critical) resistant pathogens, which urgently need new effective antibiotics (https://www.who.int/). Rapid emersion of antibiotic-resistant strains implicates the necessity of clinical management for infections. Although various treatment options have been suggested^14–19^, no effective approved option is available against *A. baumannii* and *P. aeruginosa* infections.

Active and passive immunizations are among the most promising treatment options against COVID-19^20^, *A. baumannii*^21,22^, and *P. aeruginosa* infections^23^. Effective vaccines are now available against SARS-CoV-2; however, tough challenges are remaining regarding these vaccines^24^. The safety of these vaccines is among the concerns, which should be addressed properly^25^. Recently raised infection peaks, shortly after vast vaccination programs, have revealed the ugly truth that using the vaccination approach as the sole anti-COVID-19 strategy is not sufficient in the fight against this ever-changing disease. Research on vaccine candidates against *A. baumannii* is started since 2010^26,27^ while, in the case of *P. aeruginosa*, these investigations dates back to 1980^28–31^. To date, several antigens have been nominated as vaccine candidates^32,33^. Although no clinical trials have been conducted for the vaccine candidates against *A. baumannii*, some vaccine and passive immunization candidates against *P. aeruginosa* are in phase 2/3 of clinical trials^33,34^. Recent advances in active and passive immunization against *A. baumannii*^33,35–38^ as well as recent clinical trials in the case of *P. aeruginosa*^33,34^ revealed that immunization trials are among the most promising resolves against these notorious nosocomial pathogens. However, despite numerous efforts carried out to develop effective vaccines against *A. baumannii*^36,38–45^ and *P. aeruginosa*^46–51^, no approved vaccine is yet available. An overview of these attempts revealed that single, two, and even three-component antigens could not accommodate the effective vaccine criteria. Hence, multi-component and multi-epitope antigens had been suggested^33,52^. Moreover, *A. baumannii* and *P. aeruginosa* are among the nosocomial pathogens. It is not predetermined which nosocomial pathogen would infect an admitted patient. So, to confer protection against all nosocomial pathogens (or at least the most notorious ones) several vaccinations are required. An increased number of vaccinations could result in higher costs and time of production. Thus, an all-in-one multi-epitope antigen seems to be an appropriate solution. Such antigens could be used in passive immunity as an alternative therapy against SARS-CoV-2, *P. aeruginosa,* and *A. baumannii*.

Passive immunization by antibodies that are specifically designed to mask the shared epitopes between the pathogen and its host could prevent the elicitation of autoantibodies. The highly appealing advantage of this strategy is the circumvention of autoimmune responses. The passive immunization could be only administered to hospitalized patients while the vaccination should be applied in healthy populations. Therefore, probable side effects of vaccination programs would engage a broader population in comparison to passive immunization. Antibodies have been recently used as a successful treatment option against SARS-CoV-2^24^. However, the efficacy of elicited antibodies is highly dependent on the selected antigens.

Spike glycoprotein, Outer membrane protein A (OmpA), and Outer membrane protein F **(**OprF) are the major antigens of SARS-CoV-2, *P. aeruginosa*, and *A. baumannii*, respectively. These antigens could elicit protective Abs against these pathogens^21,22,25,37,46,53–58^. Epitopes of these promising antigens have already been experimentally investigated^59–65^. Moreover, some antigen designs have been examined based on in silico analyses of spike glycoprotein, OmpA, and OprF^66–73^. However, some practical points should be considered for the design of an optimum antigen. Expression systems (prokaryotes or eukaryotes) are highly effective in the price of the final product. Expression in prokaryotic systems such as *E. coli* is cheaper than expression in eukaryotic systems^74^. Since the spike is a relatively large glycosylated antigen, it is not appropriate for *E. coli-*based expression. Several studies that used IgY or equine serum for passive immunization had expressed regions of the spike as antigens in eukaryotic systems^54,56,57,64,75,76^. It should be noted that separate expression and purification of OmpA, OprF, and spike are more expensive and time-consuming. Therefore, attempts to achieve a minimized antigen harboring non-glycosylated protective epitopes are imminently required.

The OmpA and OprF originated from Gram-negative bacteria and could be over-expressed by *E. coli*^22,37,55^. Therefore, OmpA could be employed as an appropriate scaffold to harbor non-glycosylated epitopes of the spike.

Bio- and immuno-informatics approaches have provided robust and reliable tools for various molecular biology studies^37,77–89^ from which novel vaccine design strategies are also highly benefited^66,90^.

In the present study, a strategy focused on the selection of epitopes^90^ was employed to design a novel triple-target antigen to elicit simultaneous protective antibodies against COVID-19, *A. baumannii*, and *P. aeruginosa* infections. The main idea behind the conduction of this study is the reduction of vaccine shots. A reduced number of boosters imposes less burden on the subjects, lessens the cost of mass production, and reduces the time for the manufacturing process.

Mature Outer membrane protein A of *A. baumannii* (AbOmpA) was served as a scaffold to harbor protective epitopes and peptides of AbOmpA, OprF (*P. aeruginosa*), and spike protein of SARS-CoV-2. Effective epitopes and peptides of AbOmpA and OprF were selected based on previous studies^49,59,66^. Antigenic regions of the spike including the regions involved in spike-angiotensin-converting enzyme 2 (ACE2) interaction, spike cleavage site, and membrane fusion site were selected for antigen design. Structurally consistent and/or non-protective regions of AbOmpA were replaced by protective epitopes from spike and OprF. The final construct could be expressed in a prokaryotic host and is expected to elicit protective antibodies against three pathogens. Our results revealed that the designed antigen could also overcome the emerging variants of SARS-CoV-2.

## Methods

### Workflow

In the present study, the mature OmpA of *A. baumannii* was innovatively engineered to harbor experimentally validated B-cell epitopes of spike glycoprotein (SARS-CoV-2), OmpA (*A. baumannii*), and OprF (*P. aeruginosa*) in the way that the whole protein integrity would be maintained. In this epitope-focused strategy, the most reliable epitopes of OmpA were retained; a region of OmpA which is similar to OprF_311–341_ at the sequence level was replaced. A structural approach was followed for engaging the Spike glycoprotein epitopes in OmpA; external loops and internal turns of the barrel domain of OmpA were replaced by linear B-cell epitopes of the spike. The structural analysis tools were used to delve into the detailed structure of the designed antigen and epitopic segments as engineering focal points.

### Sequences

Reference sequences including AbOmpA (accession no.: Q6RYW5)^66^, OprF (accession no.: P13794), and spike glycoprotein (accession no.: P0DTC2) were obtained from UniprotKB at https://www.uniprot.org/.

### Selection of validated epitopes

AbOmpA and OprF are well-studied antigens of *A. baumannii* and *P. aeruginosa* respectively; hence the experimentally validated epitopes of these two antigens were obtained from the related literature. The most protective epitopes of AbOmpA include “KYDFDGVNRGTRG”, “PRKLNERLSLARANSV” and “ADNKTKEGRAMNRRVFATITGSRTV”^59,66^ were considered to remain intact during the design process. And the epitope 8 (OprF_311–341_, EGGRVNAVGYGESRPVADNATAEGRAINRRV)^49^ was selected as a protective epitope from *P. aeruginosa* OprF.

The B-cell epitopes of SARS-CoV-2 spike glycoprotein were selected from the critical region of the protein, it is 437–806 encompassing receptor binding motif, furin cleavage site, and fusion peptide. The epitope prediction approach was done by four servers: BepiPred^91^ at http://tools.iedb.org/bcell/, BepiPred 2.0^92^ at http://www.cbs.dtu.dk/services/BepiPred/, SVMTrip^93^ at http://sysbio.unl.edu/SVMTriP/ and LBtope^94^ http://crdd.osdd.net/raghava/lbtope/.

The properties of the S_437–806_ sequence in favor of B-cell epitopes were assessed at http://tools.iedb.org/bcell/ including flexibility^95^, surface accessibility^96^, hydrophilicity^97^, and beta-turn secondary structure^98^.

The predicted epitopes were searched against the validated epitope library of spike glycoprotein of SARS-CoV-2 available at Immune Epitope Data Bank (IEDB) using a BLAST search. The BLAST search, as provided by IEDB (http://www.iedb.org/), was limited to positive B-cell assays; the similarity of epitope was set as 90%.

### Conservancy of the selected epitopes among various variants

The selected epitopes of the SARS-CoV-2 spike were aligned against all spike sequences of GISAID^99^ by the AnalyzeAlign tool of the COVID-19 Viral Genome Analysis Pipeline as provided by https://cov.lanl.gov/. The antibody-antigen disrupting mutations were collected from https://weilab.math.msu.edu by MutationAnalyzer^100,101^. All positions within the selected epitopes of Spike were evaluated for the existence of disrupting mutations. Additionally, the selected epitopes of the SARS-CoV-2 spike were evaluated to nominate important mutations that occurred among the most recent variants. In this regard, mutations that occurred within these selected epitopes, as reported by the Centers for Disease Control and Prevention (CDC, https://www.cdc.gov/coronavirus/2019-ncov/variants/variant-info.html, last access December 1, 2021) were considered. Additionally, reported mutations of lambda variant, were especially taken into account^102^.

### Disrupting mutations

The disrupting property of mutations in the spike glycoprotein was evaluated at https://weilab.math.msu.edu/MutationAnalyzer/. The database provides experimental data on the effect of mutations in weakening or abolishing the antigen–antibody affinity of known epitope-antibody complexes.

### Antigen design

The OmpA of *A. baumannii* served as a scaffold to present the epitopes of spike glycoprotein and *P. aeroginusa*. To note, the experimentally validated OmpA epitopes remained intact. Therefore, the final construct would contain the collection of epitopes from the three mentioned pathogens.

The signal peptide and the last 15 aa of OmpA were removed based on the previous study^66^. The sequence-based strategy determined the best matching region of OmpA with OprF_311–341_. OmpA and OprF epitope (OprF_311–341_) were aligned by ClustalW^103^ at http://www.ibi.vu.nl/programs/pralinewww/. The matched region within OmpA was replaced with OprF_311–341_.

A structural approach was employed to find regions to replace by spike epitopes. Loops (except L3) and internal turns of OmpA β-barrel were replaced by the selected peptides of S_437–806_ to present the epitopes of interest (Table 1).

**Table 1:** Loops and internal turns of OmpA β-barrel replaced by the selected epitopes of S_437-806_.

OmpA peptide	Spike epitope (SARS-CoV-2)
NNGGKDGNLTNGPELQDD	NSNNLDSKVGGNYNYLYR
GDVDGASAGAE	IYKTPPIKDFGGF
LTPW	YGFQPTNGVGYQ
LNDAL	YGFQPTNGVGYQ
KN	CNGVEGFNC
NADEEFWN	QTQTNSPRRARSV

### Construct analyses

#### Antigenicity, epitope retrieval, safety, and physicochemical properties

The antigen probability of the designed construct was evaluated by VaxiJen^104^ at http://www.ddg-pharmfac.net/vaxijen/VaxiJen/VaxiJen.html.

The designed construct sequence served as input data for BepiPred, BepiPred 2.0, SVMTrip, and LBtope to retrieve the epitopes of S glycoprotein, OprF, and OmpA predicted by these tools in the native OmpA. Allergenicity of the antigen was predicted by AllergenFP v.1.0^105^ at https://ddg-pharmfac.net/AllergenFP/ and AlgPred 2.0^106^ at https://webs.iiitd.edu.in/raghava/algpred2/. The toxicity of the construct was evaluated by ToxinPred^107^ at https://webs.iiitd.edu.in/raghava/toxinpred/protein.php in which default settings were retained.

The flexibility, surface accessibility, hydrophilicity, and beta-turn secondary structure of the designed constructs were assessed by available tools of IEDB. ProtParam server at https://web.expasy.org/protparam/ was employed to estimate some physicochemical properties of the construct such as isoelectric point (p*I*) and instability index.

### Beta-barrel OMP classification

The scaffold (OmpA) used for displaying epitopes is a known outer membrane protein. To predict whether this classification is retained after sequence replacements, the following servers were harnessed to predict mature OmpA and the construct classifications. The BOMP^108^ (http://services.cbu.uib.no/tools/bomp/) is ranking integral OMPS in five ranks (1 to 5) in which 5 revealed the most reliable, and 1 indicates the least reliable prediction^108^. HHomp^109^ (http://toolkit.tuebingen.mpg.de/hhomp) is employing an integrated beta-barrel prediction method to compare a generated profile of the Hidden Markov Model (HMM), from a query sequence, with a HMM database representing outer membrane proteins^109^. MCMBB^110^ (http://athina.biol.uoa.gr/bioinformatics/mcmbb/) scores beta-barrel outer membrane proteins as > 0 (accuracy of > 90%)^110^.

### Topology of the constructs

The topology of transmembrane proteins could be assisted in the accurate prediction of 3D structure. OMPs contain transmembrane β-strands, which could not be detected by transmembrane helix predictors. The topology of the designed construct was predicted by specialized transmembrane β-strand discriminators, viz. PRED-TMBB^111^ at http://bioinformatics.biol.uoa.gr/PRED-TMBB/ and BOCTOPUS2^112^ at http://boctopus.cbr.su.se/pred/ PRED-TMBB2^113^ at http://www.compgen.org/tools/PRED-TMBB2. PRED-TMBB is scoring a given sequence to predict whether the sequence is a beta-barrel outer membrane protein (< 2.965 are beta-barrel outer membrane). This tool provided three methods (Viterbi, N-best, and Posterior Decoding) to determine the topology of a given sequence. All the provided methods were employed for the prediction performance. The construct topology was compared to the OmpA topology.

### Structure prediction and conformational epitopes

RaptorX-Property^114^ at http://raptorx.uchicago.edu/StructurePropertyPred/predict/ was used to predict the secondary structure of the designed construct. RaptorX-Property predicts secondary structure, solvent accessibility, and disordered regions of a given protein sequence^114^. This server had been appointed as the best secondary structure predictor in an evaluation study^115^.

Several robust servers with different approaches were employed to predict the 3D structure of the designed construct. GalaxyWEB^116^ at http://galaxy.seoklab.org/, FALCON@home^117^ at http://protein.ict.ac.cn/TreeThreader/, I-TASSER^118^ at http://zhanglab.ccmb.med.umich.edu/I-TASSER/, Phyre2^119^ at http://www.sbg.bio.ic.ac.uk/phyre2/html/page.cgi?id=index, ROBETTA^120^ at http://robetta.bakerlab.org/ and RaptorX^121^ at http://raptorx.uchicago.edu/. GalaxyTBM^122^ in GalaxyWEB is a template-based modeler which builds the reliable core from multiple templates; then, it detects variable regions, such as loops, to be re-modeled by an ab initio method^122^. FALCON@home is a novel threading approach that uses remote homologous proteins as templates identified by an improved method^117^. I-TASSER uses multiple threading approaches to identify templates for iterative template-based fragment assembly simulation of full-length atomic models. This server had been ranked as the 1^st^ predictor in several community-wide CASP experiments^118^.

Phyre2 uses advanced methods to detect remote homologous for modeling a given protein. This server is ranked as a quarter 1 protein modeling tool in CASP9 and 10^119^. ROBETTA harnesses comparative modeling and de novo structure prediction methods to generate structural models^120^. RaptorX is a template-based protein structure modeling server appropriate for proteins with no close homolog in PDB^121^.

To screen the obtained models, the quality of the predicted structures was evaluated by QMEANDisCo^123^ (https://swissmodel.expasy.org/qmean/) and ERRAT^124^ (https://servicesn.mbi.ucla.edu/ERRAT/). QMEANDisCo is a single model method for the quality assessment of a predicted protein structure. This method combines statistical potentials and agreement terms with a distance constraint (DisCo) score indicating consistencies of pairwise CA-CA distances from a predicted structure with constraints of homologous structures^123^. ERRAT uses reliable high-resolution structures to show errors in a protein structure based on the statistics of non-bonded atom–atom interactions in the structure of interest^124^. Ramachandran plot of the models was delineated by PROCHECK^125^ (https://servicesn.mbi.ucla.edu/PROCHECK/).

Moreover, complying with the models and predicted secondary structure and topology of the construct were considered.

The best model was refined by 3Drefine^126^ and GalaxyRefine^127^ servers. 3Drefine refines the protein models by integrating iterative optimization of hydrogen bonds and energy minimization of the optimized model at the atomic level^126^. GalaxyRefine refines the models via rebuilding and repacking side chains followed by molecular dynamics simulation to perform overall structure relaxation. CASP10 assessment acknowledged GalaxyRefine as the most successful tool in improving the local structure quality^127^. The quality of refined models was evaluated by QMEANDisCo, ERRAT, and PROCHECK. An accurate reliable 3D structure is an essential input for the prediction of conformational B-cell epitopes in a given antigen. The best-refined model of designed all-in-one antigen was submitted to ElliPro^128^ at http://tools.iedb.org/ellipro/. This tool predicts potential linear and conformational B-cell epitopes of a given protein structure. Moreover, DiscoTope server^129^ at www.cbs.dtu.dk/services/DiscoTope/ predicting discontinuous B cell epitopes from protein 3D structures was harnessed. The method combines the calculation of contact numbers and a novel epitope propensity amino acid score of residues in spatial proximity^129^. The epitopes of interest were mapped on the best-refined 3D structure of the construct.

### Recombinant expression improvement

The protein sequence of the designed all-in-one antigen served as a query for the codon optimization tool of VectorBuilder at https://en.vectorbuilder.com/tool/codon-optimization.html to obtain optimized DNA sequence to be expressed in *E. coli*. The optimized DNA sequence was submitted to TIsigner (Translation Initiation coding region designer)^130^ at https://tisigner.com/tisigner to optimize translation initiation sites. Opening energy (mRNA accessibility), which is specific to the expression hosts, is calculated and optimized. The expression score is predicted from the minimum to maximum level (0 to 100) for the input sequence and the optimized one.

## Results

### B-cell epitopes of receptor binding motif (RBM), cleavage site, and fusion peptide regions

To determine the location of B-cell epitopes of RBM, cleavage site, and fusion peptide, different algorithms were applied. Three out of four software tools have predicted epitopes within the mentioned motives of the spike glycoprotein (Supplementary Table S1). Data in Supplementary Table S1 suggest the overlap of prediction with experimentally confirmed epitopes of OprF and OmpA. The predicted epitopes in all scenes are not completely consistent with experimental results. For instance, LBtope failed to assign the experimentally validated epitopes of OmpA. In the case of OprF, true positive results were achieved.

**Figure 1:**
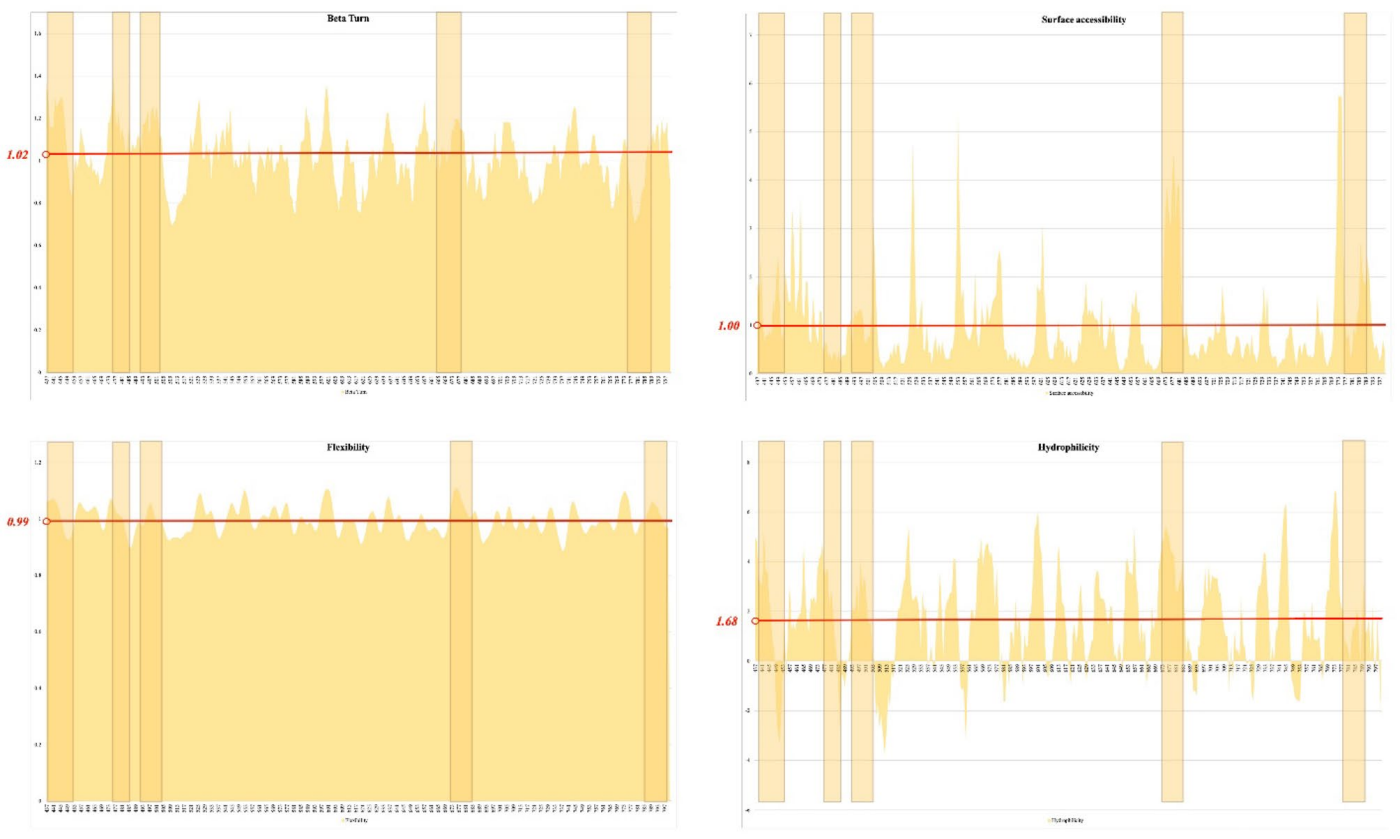
Physicochemical properties of the spike glycoprotein region (S_437–806_). Four physicochemical properties of the selected region of spike glycoprotein in favor of B-cell epitope propensity are presented by histograms. The selected regions in each histogram are shown by light brown transplant boxes; each graph is labeled by the related property. X-axes are the amino acid number and Y-axes are the score of the property.

The selected epitopes of S_437–806_ met at least one of the physicochemical properties appropriate for B-cell epitope predictions (Fig. 1). The average flexibility, hydrophilicity, and beta-turn secondary structure of S_437–806_ were 0.997, 1.678, and 1.020 respectively.

All the selected peptides matched to experimentally validated B-cell epitopes of S_SARS-CoV-2_ glycoprotein. Moreover, QTQTNSPRRARSV and IYKTPPIKDFGGF shared similarities with human peptides (the data derived from BLAST search against IEDB epitopes; see “Methods”).

### The selected epitopes of SARS-CoV-2 could be recognized in important variants

**Figure 2:**
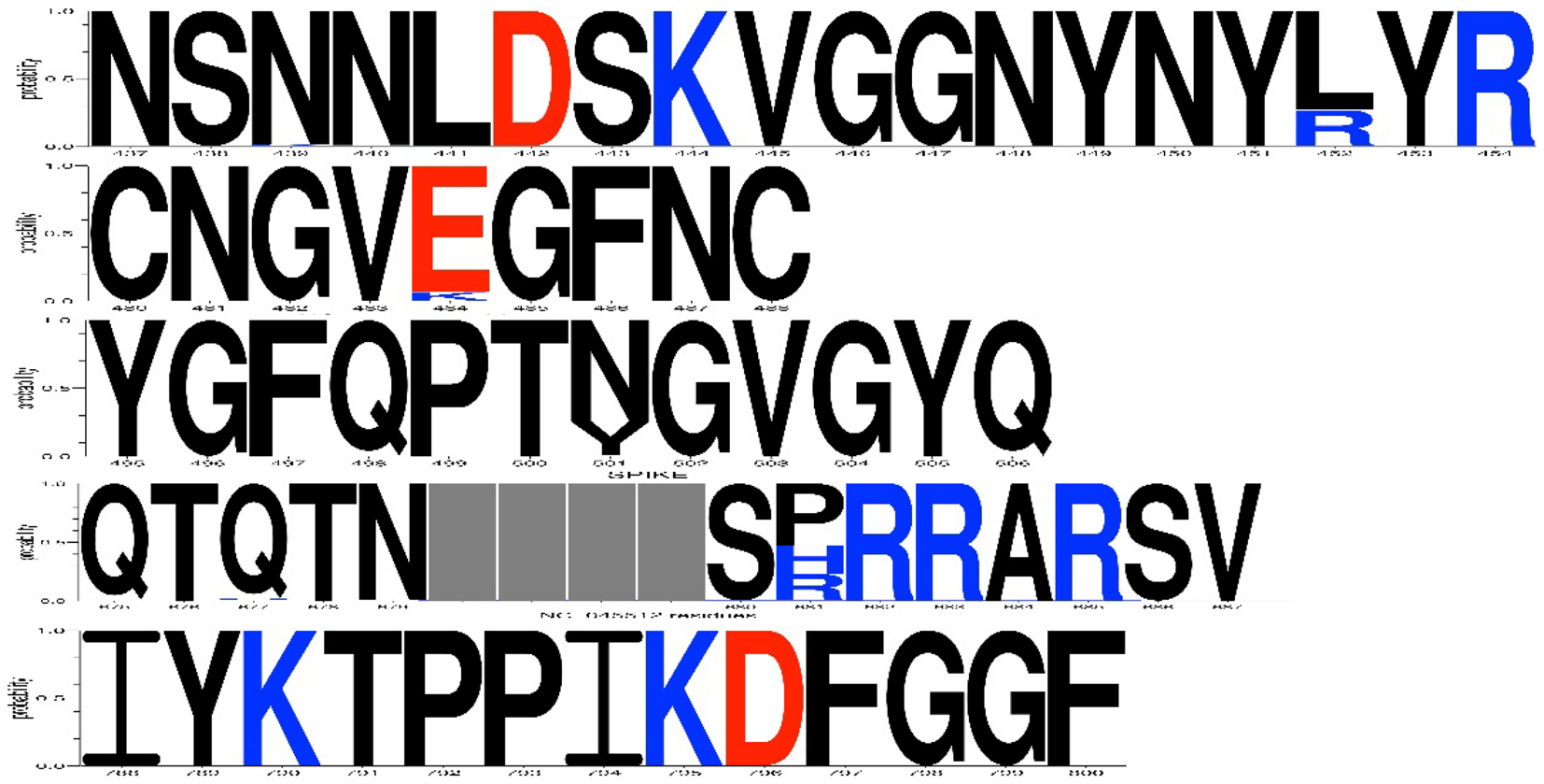
Conservancy of selected spike epitopes. The conservancy of epitopes derived from the alignment of the query epitope with all available sequences of SARS-CoV-2 spike glycoprotein is presented as the sequence logo. The X-axes are the position number of the amino acid based on the position number of the reference sequence; Y-axes are the probability. One letter amino acid symbol is stacked in each related position and the height of the symbols is proportional to the relative frequency of the residue at the respective position. The color scheme of the symbols is based on charge (positively charged amino acids are colored blue and negatively charged ones are colored red); gray is assigned to unspecified gaps in the alignment.

To precisely select the most species-inclusive epitopes the conservancy assay was done using the alignment approach. The epitopes of interest were compared to more than 81,000 submitted sequences of spike glycoprotein available at GISAID. The conservancy of epitopes was presented as sequence logos (Fig. 2). The sequence logos suggest the presence of minor variability within the selected epitopes (Supplementary Table S2). To evaluate the effect of mutations that disrupt or weaken the antibody-antigen interaction, all positions of epitopes were compared to available data. Although some disrupting mutations are observed in different variants, at least one epitope remains intact (compare Table 2 and Supplementary Table S2). Therefore, the vaccine-escape property of the virus is expected to rule out to a large extent. Moreover, a special comparison of the selected epitopes with sequences of important variants revealed a conservancy of epitopes.

**Table 2:** Selected Epitopes and mutations in emerging variants.

Epitope in wild type	Position	Mutation in Eta variant	Mutation in Iota variant	Mutation in Kappa variant	Mutation in Alpha variant	Mutation in Beta variant	Mutation in Delta variant	Mutation in Gamma variant	Mutation in Lambda variant
NSNNLDSKVGGNYNYLYR	437–454	–	L452R	L452R	–	–	L452R	–	L452Q
CNGVEGFNC	480–488	E484K	E484K	E484K	E484K	E484K	–	E484K	–
YGFQPTNGVGYQ	495–506	–	–	–	N501Y	N501Y	–	N501Y	–
QTQTNSPRRARSV	675–687	Q677H	–	P681R	P681H	–	P681R	–	–
IYKTPPIKDFGGF	788–800	–	–	–	–	–	–	–	–

The conservancy of the 5 selected epitopes was analyzed among the various important variants of SARS-CoV-2. Amongst, IYKTPPIKDFGGF was a consensus epitope with no mutation. more details are provided in Table 2.

### The designed construct is harboring conserved epitopes of OmpA, OprF, and spike glycoprotein

**Figure 3:**
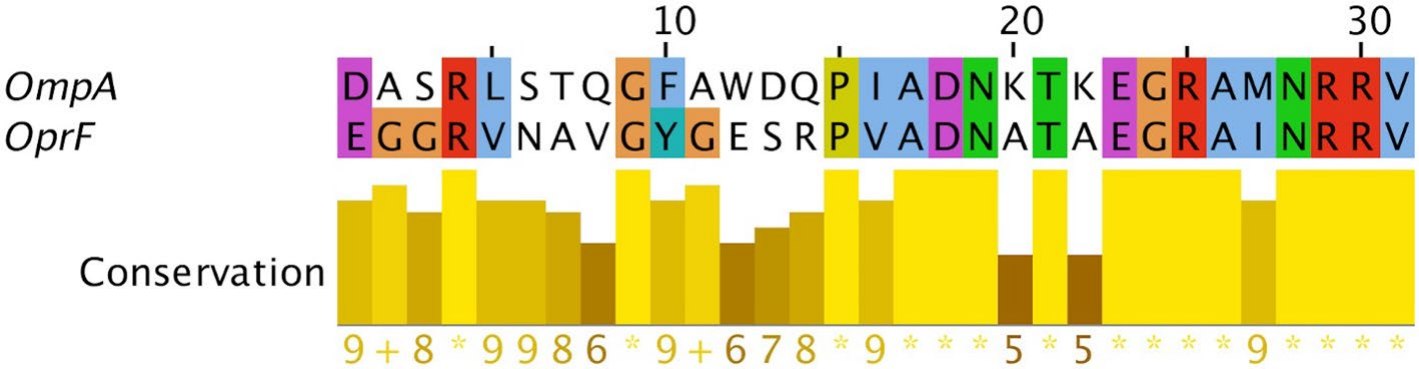
Alignment. The alignment of OmpA and OprF_311–341_. The alignment of the OmpA region (OmpA_301–331_) matched to OprF_311–341_ is presented in the upper panel and colored based on the ClustalX coloring scheme (31 amino acids). The lower panel illustrates the conservancy scores of each residue. The scores are from one to 10 to show the conservation level (low to high, respectively). Asterisks show identical amino acids; the plus symbols show the tiny similar amino acids. The sequences are labeled by the protein names.

The alignment of the AbOmpA sequence and OprF_311–341_ from *P. aeruginosa* suggests a great level of identity between the AbOmpA and OprF_311–341_ sequence (Fig. 3). Therefore, the identical segment of OmpA (OmpA_301–331_) was replaced by OprF_311–341_.

**Figure 4:**
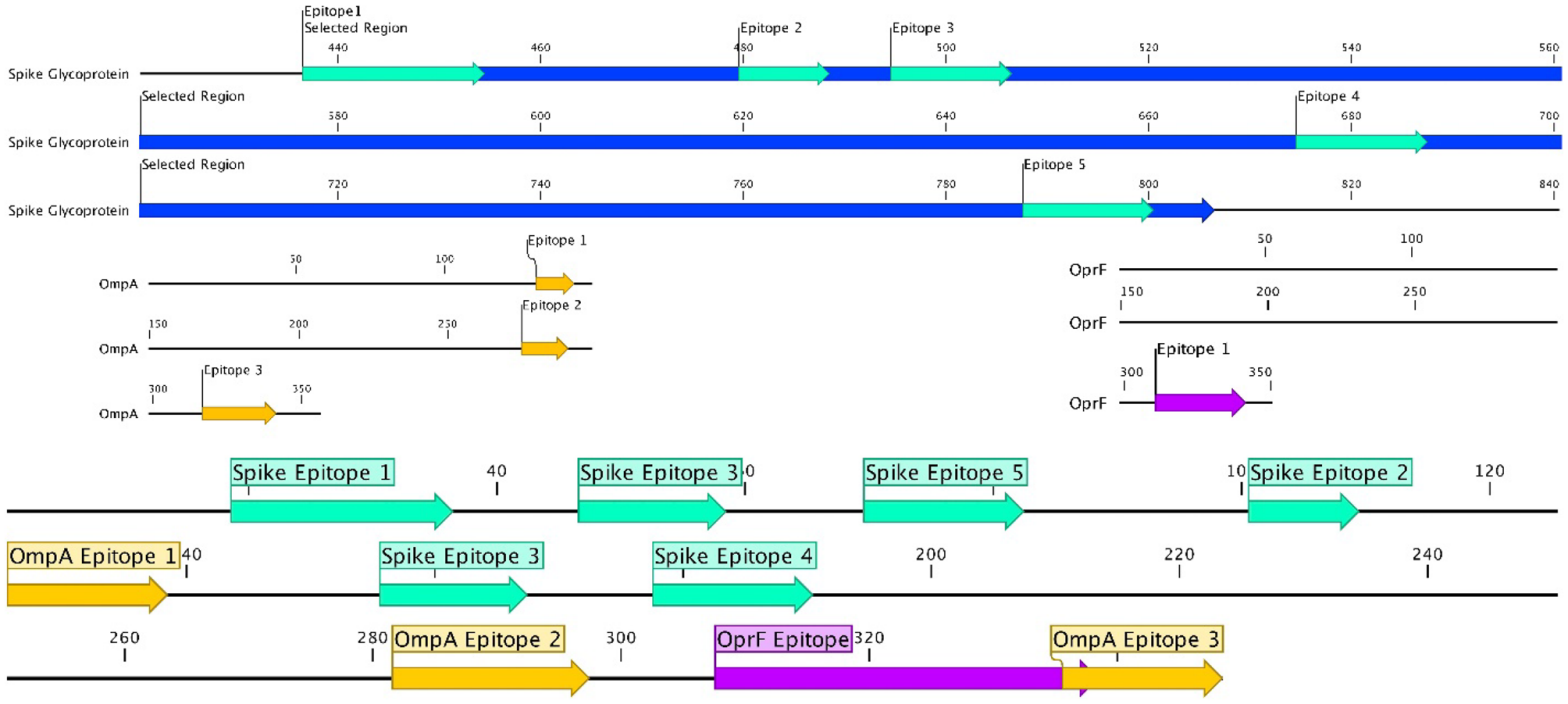
Construct schematics. The schematic representation of the engineered construct and the location of epitopes. Epitopes of different sources are shown in different colors.

Loops (except L3) and internal turns of OmpA β-barrel were replaced with antigenic regions of spike involved in its interaction with ACE2 (RBM), spike cleavage site, and fusion peptide with host cellular membrane. The final design comprised a 348 aa sequence (Fig. 4).

### Retrieval of predicted epitopes in construct with appropriate physicochemical properties

To confirm the efficiency of the construct, the novel sequence was evaluated for antigenicity, flexibility, hydrophilicity, surface accessibility, and beta-turn propensity.

**Figure 5:**
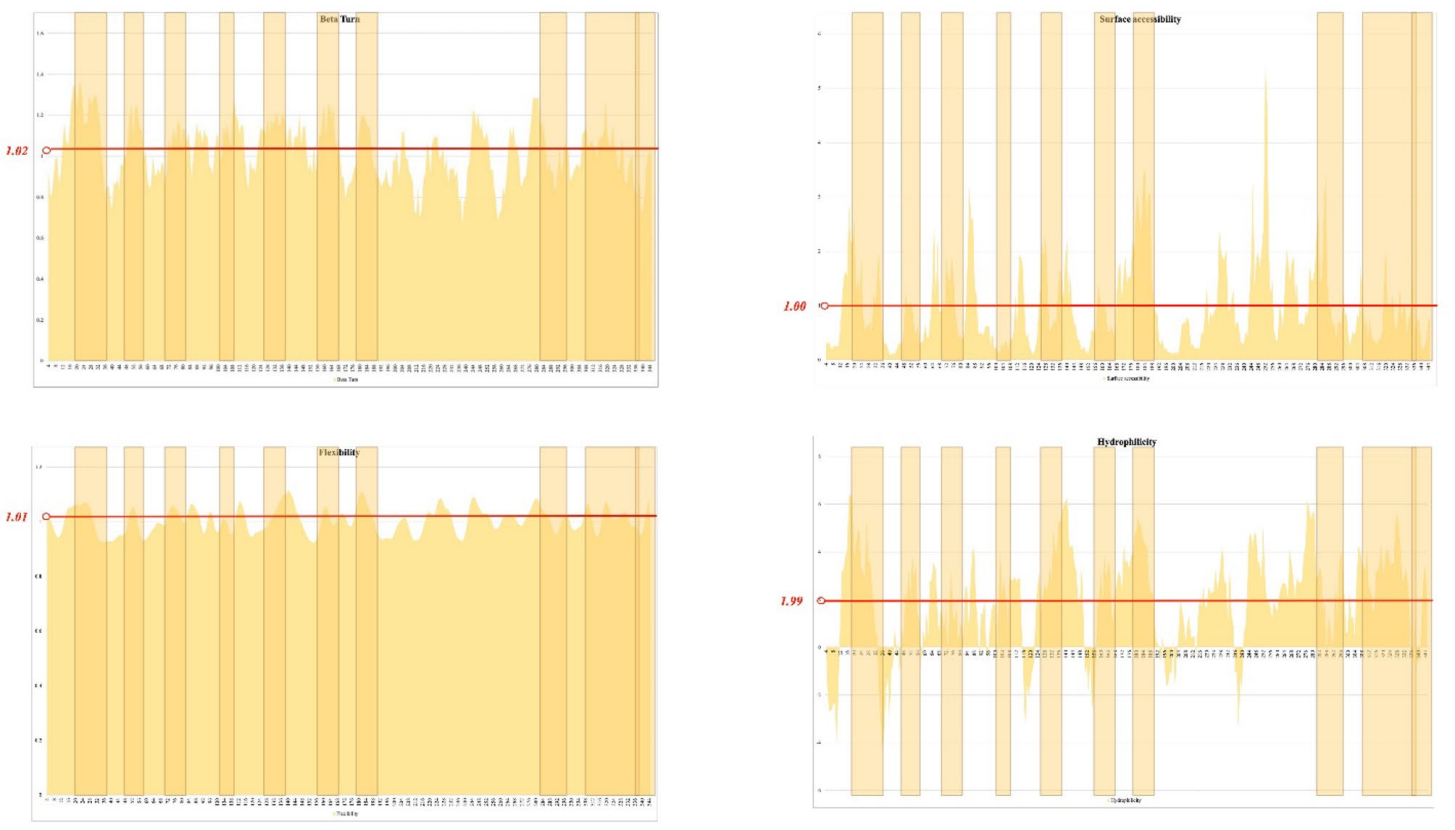
Physicochemical properties of the designed construct. Four physicochemical properties of the selected region of spike glycoprotein in favor of B-cell epitope propensity are presented by histograms. The selected regions in each histogram are shown by light brown transplant boxes; each graph is labeled by the related property. The average flexibility, hydrophilicity, and beta-turn secondary structure of the designed construct were 1.005, 1.991, and 1.023 respectively. X-axes are the amino acid number and Y-axes are the score of the related property.

The antigenicity score of the designed construct was 0.7927 while the antigenicity score of S_437–806_ was 0.5509. In the new context, the selected epitopes were flexible, hydrophile, surface accessible, and/or a beta-turn (Fig. 5). The epitopes were retrieved by epitope predictors, suggesting an accurate presenting property of the scaffold. AllergenFP predicted the construct as a non-allergen while AlgPred 2.0 hallmarked it as an allergen. The construct was characterized as non-toxic. The theoretical pI and instability index of the construct was calculated as 8.79 and 31.56 respectively. Proteins with an instability index of < 40 are classified as stable.

### Knowledge-based structural prediction of the construct

Our preliminary approach to finding the most appropriate structure for the construct sequence was to predict the structural classification, topology, and secondary structure of the construct. The perspective obtained from these predictions has paved the way for selecting, quality assessment, and confirmation of tertiary structures.

#### The designed construct was classified as an OMP

To determine the overall topology of the construct, three topological classification approaches were performed. Although one algorithm out of three did not predict the native OmpA as a ß-barrel outer membrane protein, all approaches have classified the engineered construct as a ß-barrel outer membrane protein with high scores and confidence. This reveals the maintenance of the whole structure and the accuracy of structural replacements. Therefore, the overall integrity of the beta-barrel is expected from engineered OmpA.

#### The novel antigen is composed of an 8-stranded beta-barrel and a globular C-terminus domain

To get insight into the overall structure of the modified sequence (it is the construct) the topology of the structure was evaluated. PRED-TMBB assigned the designed construct as a beta-barrel outer membrane and scored a value of 2.903. This score was 2.885 for the mature sequence of OmpA. The complete outputs of the beta-barrel predictors are summarized in Supplementary Table S3. As is evident in the Table, the sequence is of a beta-barrel outer membrane protein.

### Secondary structure

The secondary structure of the designed construct was predicted by two different tools.

The secondary structure components of the designed construct were 11% alpha-helix, 50% beta-strand, and 38% coil. The consistency of the predicted secondary structure with the predicted topology of the construct was visually inspected. The comparison implies the conformity of structural elements with different prediction approaches. This would be a guideline for assessing the quality and accuracy of tertiary structure.

### Tertiary structures

Overall, 31 3D models were the result of structural prediction of six different approaches.

Along with the consistency of topology and secondary structure components with the predicted structure, the overall quality of the structures was also investigated. The most qualified structure that gained the best scores and showed the best consistency with the predicted topology and secondary structure was selected for further analysis.

**Figure 6:**
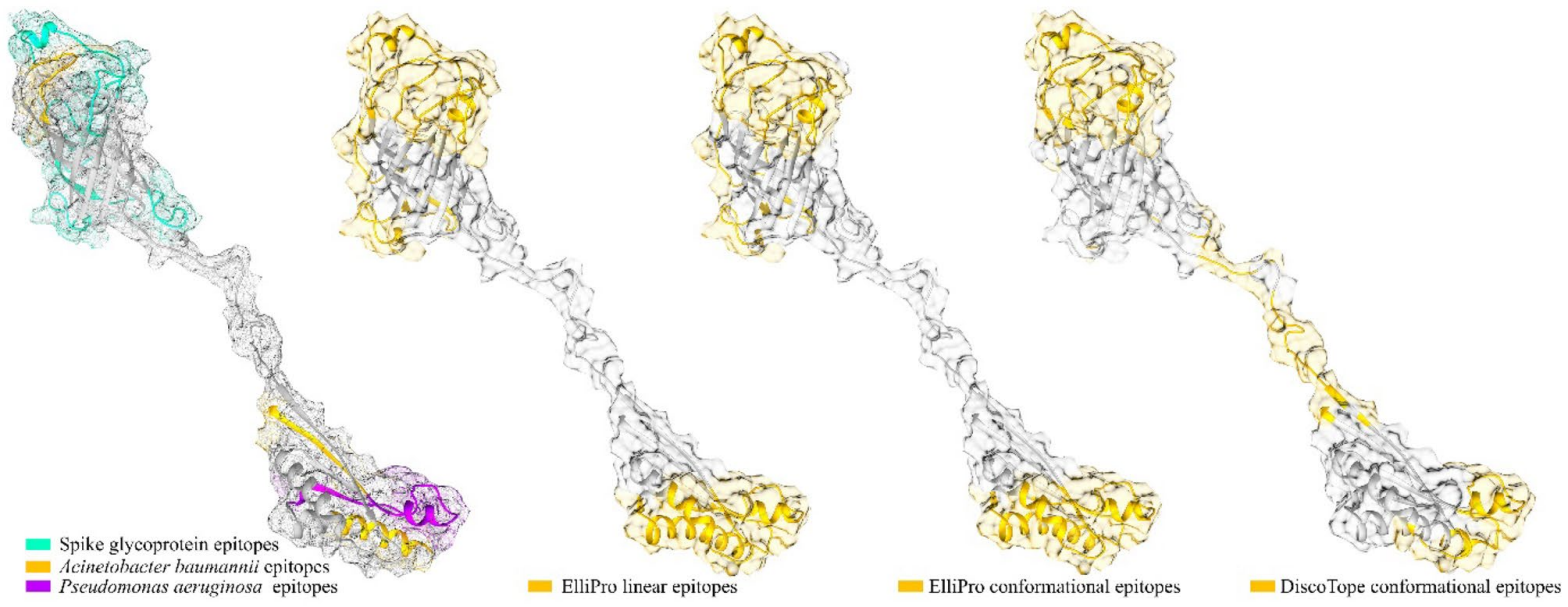
Construct epitopes. The tertiary structure of the engineered construct and the B-cell epitopes. Cartoon representation of the engineered construct is presented by gray color and transplant surface. The epitopes are assigned by colors. From left to right the first image is the construct and the embedded epitopes. Three other images show the located (predicted) epitopes of engineered construct by online servers. The color codes are shown below each image, golden ribbons are predicted epitopes. The locations of predicted epitopes in comparison to the location of embedded epitopes show the accuracy of the engineering approach. The color scheme is consistent with Fig. 4.

One out of five suggested models by GalaxyWEB was assigned as the most qualified structure. Ramachandran plot showed that 90.4% of residues in this model were in the favored region. (A qualified model typically has at least 90% of its residues in the favored region. This model has undergone a refinement process by which 10 refined models were provided (Supplementary Table S4). The epitopes of interest mapped on the 3D structure were surface exposed and accessible (Fig. 6).

### Optimized sequence for recombinant expression in *E. coli*

The protein sequence of the designed all-in-one antigen was submitted to the codon optimization tool of VectorBuilder to obtain an optimized DNA sequence for *E. coli* expression. An optimized DNA sequence with GC content of 55.49% and codon adaptation index (CAI) of 0.93 was suggested (Supplementary Fig. S1). Opening energy (mRNA accessibility) of translation initiation sites in the obtained DNA sequence was optimized by TIsigner to be expressed in *E. coli*. The expression score was predicted 30.3 and 93.24 for the input sequence and the optimized one respectively. The opening energy was calculated as 14.55 and 8.13 kcal/mol for the input sequence and the optimized one respectively.

## Discussion

The research community has faced various problems in implementing treatments against SARS-CoV-2, including antigen design, vaccine escape, weak immunity, multiple vaccine shots, and manufacturing costs, to name but a few. A dramatic increase in hospitalization during the COVID-19 pandemic would consequently lead to an upsurge in exposure to nosocomial infections. Among various opportunistic pathogens, *A. baumannii* and *P. aeruginosa* are the most notorious ones. While these bacterial pathogens are resistant to most available antibiotics, vaccination or passive immunization seem to be the best therapeutic options. The design of a multi-pathogen covering antigen to be used in passive or active immunization could be a proper solution for various challenges raised following recent events. Bacterial outer membrane proteins hold promise for the presentation of foreign peptides (epitopes in the present case)^131^. This approach would require the highly intelligent design of antigens by the implementation of prominent methods such as in silico tools. As is the case in the present study, in silico tools have been used to collect, define structures, and engineer AbOmpA to accommodate and present the foreign epitopes, derived from *P. aeruginosa* and SARS-CoV-2 antigens.

Vaccine design and development require prevailing over some immunological complications. One of the most pronounced concerns is the safety and long-term duration of protection a vaccine can provide^25^. The other challenging complication of vaccination is triggering autoimmune responses^132^. Antibody-dependent enhancement (ADE) is a further concern in which binding between the Fc receptor and the Fc region of IgG is one of the main mechanisms of this phenomenon^133^. In this regard, passive immunization by polyclonal antibodies such as IgY and equine serum raised against protective epitopes could be considered a safer alternative against various viral and bacterial infections^24,134^. As an additional advantage, the passive immunization protocol is less complicated in terms of the required criteria for clinical approval. Vaccination should be performed in healthy individuals while passive immunotherapy could be only administered to hospitalized patients. So, in comparison with vaccination, a smaller population could be affected by the probable side effects of passive immunization.

The first and foremost step in the development of passive and active immunization is the introduction of an appropriate antigen. Rational antigen designs could be harnessed to circumvent these limitations. Viruses can evade the host immune responses using unprecedented mechanisms. Some potential routes are ADE, antigen glycosylation, and altering immune-dominant epitope presentation. ADE is well-characterized in SARS-CoV-2 infections^102,135,136^. Viruses use glycosylation or removal of glycans to escape the host immune system^137^. S glycoprotein of the SARS-CoV-2 is a heavily glycosylated protein, which could induce neutralizing Abs. Therefore, coronaviruses most likely invoke this mechanism to evade host immunity^53^. The introduction of immuno-dominant non-neutralizing epitopes is another mechanism by which viruses deceive the host immune system. Epitope-based antigen design is an apt strategy to overcome these immune escape mechanisms^137^.

Although in silico tools could predict the potential B-cell epitopes with high accuracy, existing experimental confirmations for these predictions would significantly contribute to the confident selection of the most effective epitopes from the S glycoprotein. For a deterministic selection of neutralizing epitopes, a specific molecular mechanism of virus pathogenesis was taken into account. Thus, peptides involved in spike-ACE2 interaction, spike cleavage, and fusion with the host cell membrane were selected. The selected peptides were partially or completely overlapped with the neutralizing epitopes. The receptor-binding motif (RBM) of the receptor-binding domain (RBD) includes all residues involved in spike-ACE2 interaction. An in silico study revealed that 3 loops and 2 sheets are encompassing all residues involved in this interaction^138^. Therefore, the loops including these residues were selected to target the spike-ACE2 interaction. The fusion peptide of the spike glycoprotein is also known to induce neutralizing antibodies^53^. It contains a glycosylated asparagine residue within its sequence. Expression of this region in prokaryotic hosts would result in non-glycosylated protein. So, the antibodies produced against this recombinant protein could likely not be able to recognize the glycosylated region in the spike of SARS-CoV-2. Moreover, a glycosylated asparagine residue would mask the peptide from immune vision. Therefore, to target the fusion peptide without any immunity masking, the “NFSQIL” sequence was removed from the fusion peptide.

Due to the selective pressure and error-prone amplification of the coronavirus genome, mutations in its epitopes have become inevitable. Hence, numerous variants have been reported since the early stages of the pandemic and the emergence of new variants is ongoing. A high rate of mutations could potentially result in weakening or even abolishing the immunization effects. To overcome this challenge, our construct harbors five peptides from the spike protein. Among these peptides, at least two peptides are conserved in wild-type and other variants of the spike protein. Thus, it is expected that specific antibodies raised against the designed antigen would provide productivity against various mutants.

QTQTNSPRRARSV epitope contains more than 91% of a motif (i.e. YQTQTNSPRRAR) suggested to be responsible for the super-antigenic activity of SARS-CoV-2. This characteristic could trigger cytokine storms in adults as well as Multisystem Inflammatory Syndrome in Children (MIS-C). Aside from its similarity to neurotoxins and a viral super-antigen, this sequence structurally resembles Staphylococcal Enterotoxin B (SEB) super-antigen^139^. Surprisingly, a monoclonal antibody developed against SEB, 6D3, could bind to PRRA. Thereby, it interferes with the enzymatic cleavage of spike and inhibits in vitro viral entry^140^. QTQTNSPRRARSV also harbors a peptide that is identical to the human proteome. This peptide is suggested to be involved in the induction of autoimmune responses^132^. Therefore, this epitope should be removed from antigens designed as vaccine candidates; however, antibodies developed against this epitope could act as a Swiss army knife for passive immunization against SARS-CoV-2. In addition to the neutralization of SARS-CoV-2 and prevention of its super-antigenic activity, these antibodies could inhibit autoantibody triggering by epitope masking^141^.

It has been suggested that the production of high-potency neutralizing antibodies against RBM is affected by the weak presentation of MHC-II binders in this region of SARS-CoV-2 spike glycoprotein^142^. In this regard, the incorporation of linear B-cell epitopes within a potent antigen harboring strong MHC-II binders could assure robust induction of antibodies against the B-cell epitopes of interest.

OmpA and OprF are highly conserved antigens of *A. baumannii* and *P. aeruginosa*, respectively^21,143^. The inclusion of experimentally validated B-cell epitopes from these two orthologous OMPs could assure the elicitation of protective antibodies against *A. baumannii* and *P. aeruginosa*. OmpA is one of the most promising antigens of *A. baumannii*, which triggers high titers of protective antibodies^21,22,37^. It had been demonstrated that recombinant OmpA purified in denaturing conditions could also raise protective antibodies against *A. baumannii*. This property was attributed to linear B-cell epitopes of OmpA^22,37^. All epitopes of the designed construct were validated linear B-cell epitopes. The similarity of these epitopes with human peptides is less deleterious in passive immunization. It has been demonstrated that epitope masking with passively administered epitope-specific IgG could suppress IgG production against the given antigen^141,144^. Hence, passive immunization by specific antibodies raised against the all-in-one antigen could suppress autoimmune antibody responses against epitopes shared with human peptides. Hence, these similarities could be considered an advantage.

Antibodies raised against the linear B-cell epitopes in the new context (i.e. the designed construct), could recognize these epitopes in the original antigens (OmpA, OprF, and spike). Hence, the all-in-one antigen could be purified in denaturing conditions. Several multi-epitope antigens have already been designed against SARS-CoV-2. Predicted B and T cell epitopes of these multi-epitope antigens were fused by various repeats of known peptide linkers^68–72^. It has been demonstrated that increasing the copy number of a given sequence could enhance the specific antibody responses^145–147^. Therefore, increasing the repeats of spacers (linkers) is deleterious to misspend antibody responses. Several recombinant antigens, harboring epitope 8 (OprF_311–341_) as a protective region, are in various phases of clinical trial studies (reviewed in^23^). The OprF_311–341_ epitope shares identity with an immunodominant epitope of OmpA in *P. aeruginosa*. OmpA has a nuclear localization signal (NLS) at the C-terminal domain, which confers the cytotoxicity for this antigen. K_320_ and K_322_ are located at the NLS and substitution of these residues by Alanine could decrease OmpA cytotoxicity^148^. Interestingly, these substitutions have naturally occurred in the OprF_311–341_ epitope. Thus, replacing a similar region in OmpA with the OprF_311–341_ epitope in the designed construct decreases the antigen toxicity and enables its high dose administration. Among 4 validated epitopes of OmpA retained in the designed construct, only one epitope underwent a minor change by this replacement.

Recently, a novel OmpA-derived antigen has been designed in which the last 15 residues of CTD-OmpA were removed from the designed antigen. This region was undesirable for its antigenicity and B-cell epitope properties (hydrophilicity and flexibility). Moreover, K_320_ and K_322_ located at the NLS were substituted by Alanine, and loop 4 (NADEEFWN) of the 8-stranded barrel (in the two-domain conformer of OmpA) was replaced by loop 3 (YKYDFDGVNRGTRGTSEEGTL)^66^. It has been suggested that the designed antigen is a more antigenic and less toxic immunogen. In the current design, residues of loop 4 were replaced by a spike epitope. The last 15 residues of CTD-OmpA were removed.

High epitope density could significantly enhance the antigenicity and immunogenicity of the antigen of interest^145–147,149^. This criterion was met by the number of encompassed epitopes, the number of repeats for a given epitope, and the spatial and conformational density of epitopes. The designed all-in-one construct contains five peptides of spike glycoprotein condensed at the N-terminal domain (NTD) of the antigen (consisting of about 200 residues). Transmembrane β-strands of the OmpA barrel could act as natural spacers for the embedded peptides of S glycoprotein in the designed construct. The structural analyses revealed that the NTD is a β-barrel presenting the epitopes exposed at both sides of the barrel in the native structure. So, the epitopes of interest are more accessible compared to tandemly fused multi-epitope vaccines. One peptide was repeated at the periplasmic side of the barrel to increase its number and spatial density. Compared with multi-epitope antigens fused sequentially, it would be expected that the native structure of epitopes (at least those for OmpA and OprF) retained in the designed all-in-one construct.

Although based on in silico analyses, it would be expected that the designed all-in-one antigen could trigger robust protective and neutralizing antibodies against *P. aeruginosa*, *A. baumannii,* and SARS-CoV-2, experimental confirmations should be carried out in future studies. Safety considerations such as allergenicity, toxicity, autoimmune responses, and ADE should be assessed in addition to efficacy, in further pre-clinical and clinical studies.

## Conclusion

In silico approaches provide appropriate tools for rational design, evaluation, and engineering of a molecule that harbors immunogenic regions of target genomes. These tools are imperative for the design of multi-epitope constructs that could be useful in active and passive immunizations. OmpA from *A. baumannii* is a proper scaffold for the design of multi-epitope constructs. In the present study, OmpA was engineered to present foreign epitopes embedded within its sequence. The risk of facing nosocomial pathogens in the hospital environments, the economical and executive burdens of manufacturing multiple antigens, and the inevitability of multiple booster shot administration have made the idea of multi-target antigens an appealing one.

## Supplementary Information

Supplementary Figure S1.

Supplementary Table S1.

Supplementary Table S2.

Supplementary Table S3.

Supplementary Table S4.

## Data Availability

All data associated with this study are present in the paper or the Supplementary Information.
