# Supplementary material for: A unique antigen against SARS-CoV-2, Acinetobacter baumannii, and Pseudomonas aeruginosa
Source: Sci Rep. 2022 Jun 27;12:10852. doi: 10.1038/s41598-022-14877-5 (PMC9237110; doi:10.1038/s41598-022-14877-5)
Supplement: Supplementary file 1 — Supplementary Figure S1. [file 41598_2022_14877_MOESM1_ESM.docx]

**A unique antigen against SARS-CoV-2, *Acinetobacter baumannii,* and *Pseudomonas aeruginosa***

Mohammad Reza Rahbar^1^, Shaden M H Mubarak^2^, Anahita Hessami^3^, Bahman Khalesi^4^, Navid Pourzardosht^5^, Saeed Khalili^6^, Kobra Ahmadi Zanoos^7,^ and Abolfazl Jahangiri^8^*

Protein Sequence:


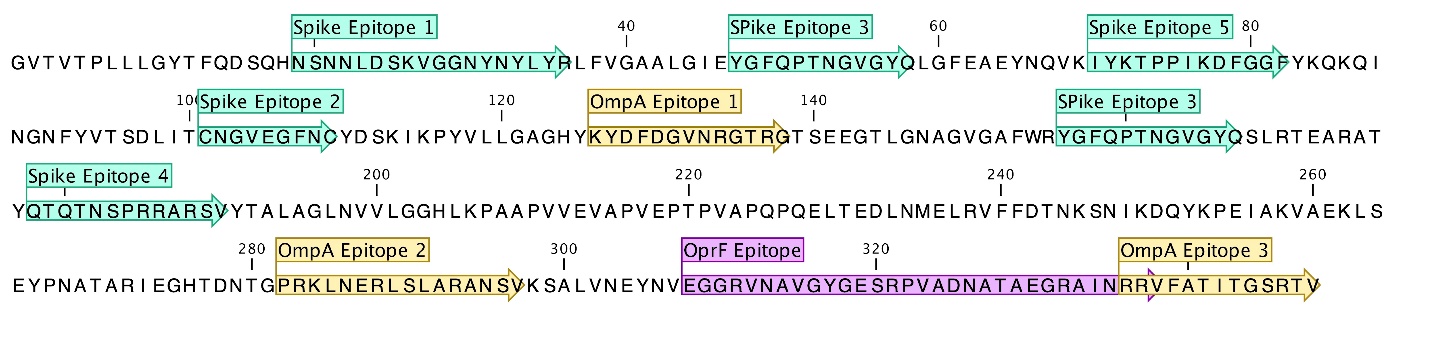


Optimized DNA sequence:

GGCGTGACCGTGACCCCGCTGTTACTGGGCTACACCTTTCAGGATAGCCAGCACAACAGCAACAATCTGGATTCCAAAGTGGGCGGCAACTATAATTACCTGTACCGCCTGTTCGTTGGTGCGGCCCTGGGCATTGAATATGGCTTTCAGCCGACGAACGGCGTGGGCTATCAGCTGGGCTTTGAAGCGGAATATAATCAGGTGAAAATTTACAAAACCCCGCCGATTAAAGATTTTGGCGGCTTTTACAAACAGAAACAGATTAATGGTAATTTTTATGTGACCAGCGATTTGATTACCTGCAATGGTGTTGAGGGCTTTAACTGCTACGATTCGAAAATTAAACCGTATGTGCTGCTGGGCGCGGGCCACTATAAATACGATTTTGATGGCGTGAATCGTGGCACCCGCGGCACCAGCGAAGAAGGCACCCTGGGTAACGCCGGAGTTGGCGCGTTTTGGCGCTATGGCTTTCAGCCGACCAATGGCGTGGGCTATCAGAGTCTGCGCACCGAAGCACGCGCGACCTACCAGACCCAGACCAACAGCCCGCGCCGCGCGCGTAGCGTGTATACGGCGCTGGCCGGCCTGAATGTGGTGCTGGGCGGCCATCTGAAACCGGCGGCGCCGGTGGTGGAGGTTGCGCCGGTGGAACCGACCCCGGTGGCGCCGCAGCCGCAGGAACTGACCGAAGATCTGAATATGGAACTGCGTGTGTTCTTTGATACCAATAAAAGCAATATTAAAGATCAGTATAAACCGGAAATTGCGAAAGTGGCGGAAAAACTGTCGGAATATCCGAATGCGACCGCCCGTATTGAAGGCCACACCGATAACACCGGCCCGCGTAAACTGAACGAACGTCTGAGCCTGGCGCGCGCCAATAGCGTGAAAAGCGCCCTGGTGAACGAATATAACGTGGAAGGCGGCCGTGTAAACGCAGTGGGCTATGGCGAAAGCCGTCCGGTTGCCGATAACGCGACCGCGGAAGGCCGCGCGATTAACCGCCGCGTGTTTGCCACCATTACCGGCAGCCGTACGGTGTAA

**Supplementary Fig. S1.** The protein sequence and the optimized DNA sequence of the designed all-in-one antigen. Epitopes of different sources are shown in different colors within the protein sequence.
