# Supplementary material for: A unique antigen against SARS-CoV-2, Acinetobacter baumannii, and Pseudomonas aeruginosa
Source: Sci Rep. 2022 Jun 27;12:10852. doi: 10.1038/s41598-022-14877-5 (PMC9237110; doi:10.1038/s41598-022-14877-5)
Supplement: Supplementary file 2 — Supplementary Table S1. [file 41598_2022_14877_MOESM2_ESM.docx]

**A unique antigen against SARS-CoV-2, *Acinetobacter baumannii,* and *Pseudomonas aeruginosa***

Mohammad Reza Rahbar^1^, Shaden M H Mubarak^2^, Anahita Hessami^3^, Bahman Khalesi^4^, Navid Pourzardosht^5^, Saeed Khalili^6^, Kobra Ahmadi Zanoos^7,^ and Abolfazl Jahangiri^8^*

**Supplementary Table S1**. Predicted linear B-cell epitopes overlapping with the favored regions of spike and the validated experimental epitopes of OprF and OmpA.

| Tool | RBM | cleavage site | fusion peptide | OprF | OmpA |
| --- | --- | --- | --- | --- | --- |
| SVMTriP | - | - | - | OprF_315-334_ | OmpA_316-335_ |
| LBtope | LDSKVG and SYGFQPTNGVG | - | - | VGYGESRPVADNATAEGRAINRR | - |
| Bepipred 1.0 | NSNNLDSKVGG, YQAGSTPCNGV and  YGFQPTNGVGYQ | QTQTNSPRRARSV | IYKTPPIKDF | GVEGGRVNAVGYGESRPVADNATAEGRA | FDGVNRGTRGTSEEGTLGNA, KLSEYPNATARIEGHTDNTGPRKL and FAWDQPIADNKTKEGRA |
| Bepipred 2.0 | LDSKVGGNYNYLYRLFRKSNLKPFERDISTEIYQAG and YGFQPT | ASYQTQTNSPRRARSVASQ | KQIYKTPPIKDFGGF | YGESRPVADNATAEGRA | KYDFDGVNRGTRGTSEEGT, NTGPRKLNERL, FAWDQPIADNKTKEGRA and RTVVVQPGQEAAAP |
