# Supplementary material for: A unique antigen against SARS-CoV-2, Acinetobacter baumannii, and Pseudomonas aeruginosa
Source: Sci Rep. 2022 Jun 27;12:10852. doi: 10.1038/s41598-022-14877-5 (PMC9237110; doi:10.1038/s41598-022-14877-5)
Supplement: Supplementary file 4 — Supplementary Table S3. [file 41598_2022_14877_MOESM4_ESM.docx]

**A unique antigen against SARS-CoV-2, *Acinetobacter baumannii,* and *Pseudomonas aeruginosa***

Mohammad Reza Rahbar^1^, Shaden M H Mubarak^2^, Anahita Hessami^3^, Bahman Khalesi^4^, Navid Pourzardosht^5^, Saeed Khalili^6^, Kobra Ahmadi Zanoos^7,^ and Abolfazl Jahangiri^8^*

**Supplementary Table S3**. Topology of the designed antigen.

| Construct topology | BOCTOPUS | PRED-TMBB2 | PRED-TMBB (Viterbi) | PRED-TMBB (N-best) | PRED-TMBB (Posterior Decoding) | Mature OmpA topology | BOCTOPUS | PRED-TMBB2 | PRED-TMBB (Viterbi) | PRED-TMBB (N-best) | PRED-TMBB (Posterior Decoding) |
| --- | --- | --- | --- | --- | --- | --- | --- | --- | --- | --- | --- |
| In 1 | 1 | 1-5 | 1-6 | 1-6 | 1-6 | In 1 | 1 | 1-6 | 1-6 | 1-6 | 1-6 |
| Tm1 | 2-11 | 6-13 | 7-13 | 7-13 | 7-13 | Tm1 | 2-11 | 7-14 | 7-13 | 7-13 | 7-13 |
| Out1 | 12-37 | 14-36 | 14-32 | 14-32 | 14-32 | Out1 | 12-37 | 15-36 | 14-36 | 14-36 | 14-36 |
| TM2 | 38-49 | 37-47 | 33-49 | 33-49 | 33-47 | TM2 | 38-47 | 37-47 | 37-47 | 37-47 | 37-47 |
| In2 | 50-56 | 48-54 | 50-54 | 50-54 | 48-54 | In2 | 48-50 | 48-50 | 48-50 | 48-50 | 48-50 |
| TM3 | 57-67 | 55-63 | 55-65 | 55-65 | 55-65 | TM3 | 51-60 | 51-58 | 51-57 | 51-57 | 51-57 |
| Out2 | 68-96 | 64-96 | 66-145 | 66-145 | 66-91 | Out2 | 61-77 | 59-81 | 58-77 | 58-77 | 58-79 |
| TM4 | 97-106 | 97-107 | 146-156 | 146-156 | 92-100 | TM4 | 78-89 | 82-90 | 78-90 | 78-90 | 80-90 |
| In3 | 107-113 | 108-113 | 157-348 | 157-348 | 101-109 | In3 | 90-96 | 91-96 | 91-92 | 91-92 | 91-92 |
| TM5 | 114-125 | 114-124 | - | - | 110-120 | TM5 | 97-108 | 97-107 | 93-103 | 93-103 | 93-105 |
| Out3 | 126-144 | 125-144 | - | - | 121-147 | Out3 | 109-126 | 108-128 | 104-128 | 104-128 | 106-128 |
| TM6 | 145-156 | 145-156 | - | - | 148-158 | TM6 | 127-138 | 129-139 | 129-139 | 129-139 | 129-139 |
| In4 | 157-166 | 157-159 | - | - | 159-166 | In4 | 139-143 | 140-142 | 140-142 | 140-142 | 140-142 |
| TM7 | 167-177 | 160-168 | - | - | 167-177 | TM7 | 144-153 | 143-153 | 143-153 | 143-153 | 143-153 |
| Out4 | 178-191 | 169-190 | - | - | 178-190 | Out4 | 154-161 | 154-159 | 154-161 | 154-161 | 154-161 |
| TM8 | 192-201 | 191-201 | - | - | 191-201 | TM8 | 162-171 | 160-172 | 162-172 | 162-172 | 162-172 |
| In5 | 202-348 | 202-332 | - | - | 202-348 | In5 | 172-334 | 173-318 | 173-334 | 173-334 | 173-334 |
| TM9 | - | 333-340 | - | - | - | TM9 | - | 319-326 | - | - | - |
| Out5 | - | 341 | - | - | - | Out5 | - | 327 | - | - | - |
| TM10 | - | 342-348 | - | - | - | TM10 | - | 328-334 | - | - | - |
