# Supplementary material for: A unique antigen against SARS-CoV-2, Acinetobacter baumannii, and Pseudomonas aeruginosa
Source: Sci Rep. 2022 Jun 27;12:10852. doi: 10.1038/s41598-022-14877-5 (PMC9237110; doi:10.1038/s41598-022-14877-5)
Supplement: Supplementary file 5 — Supplementary Table S4. [file 41598_2022_14877_MOESM5_ESM.docx]

**A unique antigen against SARS-CoV-2, *Acinetobacter baumannii,* and *Pseudomonas aeruginosa***

Mohammad Reza Rahbar^1^, Shaden M H Mubarak^2^, Anahita Hessami^3^, Bahman Khalesi^4^, Navid Pourzardosht^5^, Saeed Khalili^6^, Kobra Ahmadi Zanoos^7,^ and Abolfazl Jahangiri^8^*

**Supplementary Table S4.** Quality of refined models.

| Tool/model | QMEANDisCo | ERRAT | Most favored region | Additional allowed region | Generously allowed region | Disallowed region |
| --- | --- | --- | --- | --- | --- | --- |
| Original model GalaxyWEB 4 | 0.49 | 84.6154 | 90.4% | 8.9% | 0% | 0.7% |
| 3Drefine 1 | 0.53 | 85.1064 | 89.0% | 10.0% | 0.3% | 0.7% |
| 3Drefine 2 | 0.53 | 82.9851 | 88.3% | 10.7% | 0.3% | 0.7% |
| 3Drefine 3 | 0.53 | 82.6471 | 88.3% | 10.7% | 0.3% | 0.7% |
| 3Drefine 4 | 0.54 | 82.3529 | 88.0% | 10.7% | 0.7% | 0.7% |
| 3Drefine 5 | 0.54 | 80.2941 | 86.9% | 11.7% | 0.7% | 0.7% |
| GalaxyRefine 1 | 0.52 | 87.2414 | 92.4% | 6.9% | 0.0% | 0.7% |
| GalaxyRefine 2 | 0.52 | 83.1081 | 92.8% | 6.5% | 0.0% | 0.7% |
| GalaxyRefine 3 | 0.52 | 80.6228 | 91.8% | 7.6% | 0.0% | 0.7% |
| GalaxyRefine 4 | 0.52 | 79.0378 | 92.1% | 7.2% | 0.0% | 0.7% |
| GalaxyRefine 5 | 0.51 | 85.4167 | 92.8% | 6.5% | 0.0% | 0.7% |
